# An exploratory pilot study on health education program to improve health literacy among female in their 20s

**DOI:** 10.1186/s13104-018-3687-9

**Published:** 2018-08-13

**Authors:** Shiho Kawata, Emiko Saito

**Affiliations:** 1 Department of Nursing, Faculty of Health and Medical Sciences, Kyoto Gakuen University, 18 Gotanda-cho, Yamanouchi, Ukyo-ku, Kyoto, 615-8577 Japan; 2 Department of Nursing Sciences, Graduate School of Human Health Sciences, Tokyo Metropolitan University, 7-2-10 Higashi-Ogu, Arakawa-ku, Tokyo, 116-8551 Japan

**Keywords:** Sex education program, Health literacy, Female undergraduate students

## Abstract

**Objective:**

Health literacy (HL) is one of the most important concepts in women’s healthcare. The low cervical cancer screening rate for young Japanese women is an urgent issue. Cervical cancer is preventable, and cervical cancer screening behavior is associated with HL. Therefore, the present study aimed to elucidate the effects of a health education program to improve HL among young female undergraduate students in Japan. Immediately after completing the program, participants evaluated their level of satisfaction with and the level of difficulty of the program, their understanding of the educational materials, and the length of the curriculum. Furthermore, 1 month after completing the program, participants evaluated their overall HL and their knowledge of women’s health, and indicated whether they had undergone cervical cancer screening.

**Results:**

Thirteen female undergraduate students in their 20s participated. All participants indicated high levels of satisfaction and understanding of the material, and an appropriate level of difficulty of the curriculum. Three participants indicated that the program was too long. All participants had improved HL and knowledge of women’s health after completing the education program, but no significant difference was observed in the cervical cancer screening rate.

*Trial registration* UMINR000036690 April 10, 2018 retrospectively registered

## Introduction

Cervical cancer is the second most common cancer affecting females worldwide, with over 445,000 new cases and 270,000 deaths in 2012 [1]. The incidence of cervical cancer in Japanese women is 16.1 per 100,000, and the rates of cervical cancer have steadily risen over the past 25 years [2]. Cervical cancer is preventable, but most women diagnosed with invasive cervical cancer have never or only rarely been screened [3]. The Ministry of Health, Labour and Welfare in Japan has been conducting screening for cervical cancer for women over 20 years of age since 2008 and recommends screening once every 2 years [4]. However, many Japanese women still never or rarely participate in such screenings. Health literacy (HL), which is the degree to which individuals have the capacity to obtain, process, and understand basic health information and services needed to make appropriate health decisions [5], is one of the most important concepts in women’s healthcare for promoting such issues.

A previous systematic review concluded that HL explains cervical cancer screening behaviors [6]. Another study found that HL was low among Japanese adults [7], suggesting the importance of improving HL for Japanese people.

Education for the primary prevention of cervical cancer focusing on reducing the risk of human papillomavirus infection and transmission (along with other sexually transmitted infections [STIs]), tailored appropriately to age and culture, is currently provided to sexually healthy boys and girls [8]. However, fewer opportunities for sex education are provided to students after graduating from high school, and most such education in high school is inadequate [9]. Consequently, it is important for Japanese women in their 20s to receive sex education including HL. The aim of the present study was to examine the effects of a health education program to improve women’s HL among female undergraduate students in Japan.

## Main text

### Methods

#### Research design and participants

This study utilized a one-group pre-test/post-test quasi-experimental study design. The participants were recruited using posters placed in universities in Kyoto, Japan. The inclusion criteria were: (i) female undergraduate student; (ii) aged 20 years or older; and (iii) not a nursing, medicine, or pharmacy student. Data were collected from December 2014 to March 2015. The sample size was set to approximately 10 participants with reference to proceeding pilot studies of program development for cancer prevention [10, 11] and for healthy women [12, 13].

#### Intervention program

This program aimed to improve education for the prevention of cervical cancer, to reduce the risk for STIs, and to improve women’s HL among female undergraduate students. The program was developed with reference to previous studies using skills training for condom use [14, 15] to improve HL education by using jargon-free communication, pictures to clarify concepts, and confirmation of participants’ comprehension via the “show-me” or “teach-back” method) [16] based on the opinions of female nurses and undergraduate students. The contents of the intervention program are shown in Table 1. The program consisted of a group lecture and a demonstration (total 50 min) to provide knowledge regarding the following: (i) using a basal body temperature thermometer and a vaginal discharge model to understand one’s own menstrual cycle; (ii) using gynecological disease cases (e.g., endometriosis and STIs) to learn about recent trends in and the prevention of gynecological diseases; and (iii) using one’s own smartphone to access health resources and worksheets regarding gynecological examinations and consultations. The teaching materials used in the program were created using Microsoft PowerPoint® 2013 (Redmond, WA, USA). We also considered that audiovisual aids would allow us to watch the reactions of the participants during lectures. The literature review regarding HL and women’s health suggested that readability, layout, and design were key components in developing effective printed materials [17], so the illustrations used in the teaching materials and worksheets were done by a professional illustrator. The group lecture and demonstrations were conducted by a researcher and a public health nurse after providing an explanation of the study purpose and methods; both took place in the conference room of a public facility in Kyoto, Japan. The public health nurse had previous experience in maternal and child health practice.

**Table 1:** Contents of sex education program to improve HL

Contents	Materials
1. Knowing my body (20 min)	Slides delivered with presentation software A basal body thermometer and graph A vaginal discharge model Using one’s own smartphone A worksheet simulating a gynecological exam
Basal body temperature	
Menstrual periods	
Vaginal discharge	
2. Preventing gynecological disease (20 min)	
Sexually transmitted infections	
Communication about condom use with partners	
Cervical cancer screening	
3. Gynecological exams and consultations (15 min)	
Overview of gynecological examination	
How to select female health information	

*HL* health literacy

#### Evaluations

We conducted an anonymous questionnaire survey and managed the data using concatenate numbers. Questionnaire items consisted of age, gynecological history, menstrual cycle, sex education history, and cervical cancer screening history. Test of knowledge for women’s health was used to measure knowledge of women’s health: the potential score range for this test was 0–20 developed by the authors in reference to previous studies on women’s health [15, 18, 19]. The level of satisfaction with the program, levels of difficulty and understanding of the educational materials and time required for the program were evaluated [20]. The main outcomes were HL score and percentage of cervical cancer screening behavior at 1 month after completing the program:

- HL: HL scale for women in their 20s and 30s [21], in reference to a previous study on the development of an HL scale for Japanese [22], composed of 21 variables (score range, 21–84) organized into four subscales. The internal consistency between the four subscales varied (Cronbach’s α, 0.75–0.83).
- Percentage of cervical cancer screening behavior: Whether the participants had attended a cervical cancer screening examination (Y/N) at 1 month after completing the program.

#### Statistical analysis

The data were analyzed using SPSS 23.0 for Windows (SPSS, Chicago, IL, USA). Statistical significance was set at *P *< 0.05.

The results were analyzed by calculating basic statistics and pre-/post-comparisons of HL variables and knowledge using Wilcoxon’s signed-rank test. Comparisons of the pre-/post-percentage of cervical cancer screening behavior were performed using McNemar’s test, and the free descriptions were analyzed using content analysis.

#### Ethical considerations

Participants were informed of the study aims and methods and were assured that their participation would be voluntary and that every effort would be made to protect their privacy. We provided all materials used in the program to the participants.

### Results

A total of 13 female undergraduate students (mean age, 20.7 years; standard deviation, 0.4; age range, 20–21 years) participated in the present study (Table 2).

**Table 2:** Characteristics of the participants (n = 13)

Characteristics	Mean (SD) or n (%)
Age (years)	20.7 (0.4)
Gynecological history	
Yes	4 (30.8)
No	9 (69.2)
Menstrual cycle	
Regular	4 (30.8)
Irregular	9 (69.2)
Sex education history	
Elementary school	1 (7.7)
Junior high school	2 (15.4)
High school	8 (61.5)
University	2 (15.4)
Cervical cancer screening history	
Yes	0 (0.0)
No	13 (100.0)

*SD* standard deviation

All participants replied that their level of satisfaction with the program curriculum was either “very satisfied” or “satisfied”, that the level of difficulty was “just right”, and that the gynecological and educational materials were either “fully understood” or “understood”. However, one participant replied that program’s presentation slides were “difficult to see”, and three participants replied that the program was “long”. Many participants’ felt that the program contents “observing vaginal discharge” and “undergoing a cervical cancer screening” were useful in their daily life, and the impressions (free comments) of the program included statements such as “I had found it difficult to ask about STIs, so I depended on information from the Internet” and “I now understand that women’s health is important for a happy life”.

HL and women’s health knowledge were higher after the group lecture for all participants compared with before, but no significant differences were seen in pre-/post-percentage of cervical cancer screening behavior (Table 3).

**Table 3:** Effects of the program to improve HL and cervical cancer screening behavior

Variables	Pre-test (n = 13)	Post-test (n = 13)	*P*-value
HL scale^a^ (total)	58 (56–63)	68 (59–79)	0.001
Subscales			
Women’s choice for adopting health information and practice	24 (23–26)	28 (27–30)	0.003
Knowledge of female body	15 (14–16)	17 (15–20)	0.004
Self-care during menstruation	13 (10–15)	15 (13–17)	0.003
Sexual discussion with partner	6 (4–7)	8 (6–8)	0.023
Test of knowledge for women’s health^a^	16 (13–18)	18 (17–19)	0.003
Cervical cancer screening behavior^b^			
Yes	0 (0.0)	4 (30.8)	0.125
No	13 (100.0)	9 (69.2)	

*HL* health literacy

^a^Wilcoxon’s signed-rank test; data are expressed as median (interquartile range)

^b^McNemar’s test and this question were conducted at 1 month after completing the program

### Discussion

The results of the present study showed that female undergraduate students had a high level of understanding and satisfaction regarding a health education program designed to improve HL. An earlier pilot study for women was considered feasible and acceptable if more than 75% reported being satisfied with its contents [23]. Using easy-to-understand electronic and printed materials can advance knowledge and understanding and lead to improved cancer screening rates [24]. In brief, this program is considered feasible and acceptable for female undergraduate students. In addition, it was found that participants urgently need more sex education and want to acquire more knowledge regarding gynecological diseases. Some proceeding studies in Asia reported that unmarried women, including female university students, have few opportunities regarding education for cervical cancer and the prevention of STIs, and that there is a shortage of knowledge on cervical cancer and STIs, including risk behaviors [25, 26]. We suggest that the program provides a good opportunity for sex education and to improve HL among female undergraduate students. Only three participants (23.1%) felt that the program was long. About 70% of Japanese university students have a part-time job to support the cost of living [27]. The present program was conducted in the evenings after the end of regular classes, a time at which such students may be busy. Therefore, securing time to attend a sex education program can be difficult. In the future, it will be necessary to shorten the length of the program and offer it at a time that is more accommodating to student schedules (e.g., during health checkups).

The results showed that this program improved HL and women’s health knowledge among female undergraduate students. Some previous studies on HL and cervical cancer knowledge reported finding a relationship between cervical cancer and screening behaviors [6, 25, 28]. However, our results did not show any significant difference in the number of examinations after 1 month, so this program may not be effective in promoting participation in screening for cervical cancer. Our results suggest that the timing of evaluation of cervical cancer screening behavior was early, and thus, a methodological review is necessary.

A systematic review concluded that there were a number of barriers to participation in cervical cancer screening examinations, such as inaccessible clinics and inconvenient locations and appointment times [29]. The low cervical cancer screening rate among women in Japan is an urgent issue, and a health care system that provides unmarried women with access to such screenings without causing a psychological or physical burden is needed. As the average age at first marriage for Japanese women (31.1 years as of 2015) is increasing [30], we believe that the number of sexually experienced unmarried women is also increasing. Therefore, it is important to improve sex education tailored as appropriate to age and culture. In the future, it will be necessary to further consider the length and duration of the present program in greater detail. In conclusion, to the best of our knowledge, this is the first study in Japan to focus on improving HL in relation to women’s health. The findings are expected to improve HL in relation to women’s health among young Japanese women.

## Limitations

This study used a one-group pre-test/post-test quasi-experimental design. A study that sets a sample size with reference to the HL scores from the present study and compares HL scores with a different group needs to be conducted. The study participants were female undergraduate students likely to be interested in women’s health, but support is needed for all women, not only those interested in women’s health. Some unknown factors affect HL. Few HL studies in relation to nursing in Japan have been conducted, and some factors related to HL remain unclear; therefore, unknown related factors other than the factors used in the present study could have affected the results.

## Abbreviations

HL: health literacy
STIs: sexually transmitted infections
